# How to Save Children

**Published:** 1874-12

**Authors:** 


### HOW TO SAVE CHILDREN.

Some time ago we read an article in a popular weekly journal under the very promising caption of "How to Save Children." We determined that we would take early occasion to protest against its teachings. We know that a large per-centage of the children in this country are being destroyed or made invalids for life through the observance of such rules as are contained in the article alluded to.

The writer mistakably attributes anything like restlessness or crying to over-feeding. The truth is, that in a majority of cases the crying is caused by want of proper food, or by thirst. Infants should have water almost every hour in the day. He asserts:

"A child's stomach needs rest." Almost every child has heard that nonsense repeated when stimulated by a keen appetite to ask for more than his usual allowance of food. Now the stomach don't require any such thing. Rest for the stomach would soon consign the body to its everlasting rest. This *rest* for the stomach is sweeping away thousands of children every year, even from the homes of wealth and abundance. No intelligent person will claim that the heart, lungs, or blood require rest. Their action is involuntary, and with them rest means death. Now the action of the stomach is also involuntary, and the life of all the other organs depends on its action. It is the source from which all vital force emanates. Remove the fuel from the boiler, and the engine soon stops. Supply the furnace with poor or insufficient fuel, and the movements of the machinery will be feeble and irregular. If "starvationists" were to apply these rules to their horses, they would be ashamed to be seen driving them, after a few days of such abuse. Has anybody ever seen any living creature, old or young, in any way injured by over-indulgence in good, wholesome food? We are told that infants should be fed with great regularity, and not oftener than once in three hours. Did it ever occur to anybody, that with an infant all the operations of the system are much more rapid than with adults, and that consequently they require food oftener.

It is unfortunate for suffering humanity that there are so many people who presume to write fluently upon subjects they know nothing about. It is still more unfortunate that there are so many who pin their faith to anything and everything they see in print. It is safest to set aside all rules, and trust to the judgment and good sense of mothers and nurses of experience in the care of children. Abstain from giving soothing syrups and other poisons. When children are sick, nine cases in ten will recover much sooner with good nursing, than by resorting to medicines. When children are old enough to walk, or sooner, let them take their places at the table with the family, and eat and drink the same as others do, not excepting a little tea and coffee, if they relish it. If parents will take as good care of their children as they do of their *other* domestic animals, children with sound health and rosy cheeks will not be exceptional, and a reasonable proportion of them will not become angels till a much later period.

